# Associations of Insulin Resistance and High-Sensitivity C-Reactive Protein with Metabolic Abnormalities in Korean Patients with Type 2 Diabetes Mellitus: A Preliminary Study

**DOI:** 10.3390/metabo14070371

**Published:** 2024-06-30

**Authors:** Yuchul Jeong, Beom Jun Lee, Wonjai Hur, Minjoon Lee, Se-Hyeon Han

**Affiliations:** 1 Department of Internal Medicine, Chungna Good Hospital, Incheon 22738, Republic of Korea; 2 St. Mary’s Best ENT Clinic, Seoul 08849, Republic of Korea; 3 Department of Internal Medicine, Sejong General Hospital, Bucheon 14754, Republic of Korea; 4 Department of Internal Medicine, BS General Hospital, Incheon 23037, Republic of Korea; 5 Department of Companion Animal Industry, College of Health Science, Honam University, Gwangju 62399, Republic of Korea

**Keywords:** diabetes mellitus, type 2, insulin resistance, C-reactive protein, liver diseases, metabolic syndrome, albuminuria

## Abstract

We conducted this single-center, retrospective, cohort study to examine whether insulin resistance (IR) and high-sensitivity C-reactive protein (hsCRP) have a relationship with metabolic abnormalities in patients with type 2 diabetes mellitus (T2DM). In a total of 3758 patients (*n* = 3758) with T2DM, we analyzed medical records and thereby evaluated their baseline characteristics such as age, sex, duration of T2DM, systolic blood pressure (SBP), diastolic blood pressure (DBP), waist circumference, body mass index (BMI), visceral fat thickness (VFT), fasting plasma insulin levels, C-peptide levels, glycated hemoglobin (HbA1c), fasting plasma glucose (FPG), postprandial plasma glucose (PPG), homeostatic model assessment of insulin resistance (HOMA-IR), homeostatic model assessment of β-cell function (HOMA-β), aspartate aminotransferase (AST), alanine aminotransferase (ALT), total cholesterol (TC), triglyceride (TG), high-density lipoprotein (HDL), low-density lipoprotein (LDL), albuminuria, intima-media thickness (IMT) and hsCRP. The patients were stratified according to the tertile of the K index of the insulin tolerance test (KITT) or hsCRP. Thus, they were divided into the lowest (≥2.37), middle (1.54–2.36) and highest tertile (0–1.53) of KITT and the lowest (0.00–0.49), middle (0.50–1.21) and highest tertile (≥1.22) of hsCRP. Moreover, associations of KITT and hsCRP with metabolic abnormalities, such as steatotic liver disease (SLD), metabolic syndrome (MetS), albuminuria, diabetic retinopathy and carotid atherosclerosis, were also analyzed. There was a significant positive correlation between the prevalence of SLD, MetS, albuminuria and diabetic retinopathy and KITT (*p* < 0.001). Moreover, there was a significant positive association between the prevalence of SLD, MetS and albuminuria and hsCRP (*p* < 0.001). In conclusion, our results indicate that clinicians should consider the relationships of IR and hsCRP with metabolic abnormalities in the management of patients with T2DM. However, further large-scale, prospective, multi-center studies are warranted to confirm our results.

## 1. Introduction

Since the introduction of a Westernized dietary habit, accompanied by reduced physical activities, in Asian countries, there has been an increase in the prevalence of lifestyle diseases, such as central obesity, type 2 diabetes mellitus (T2DM), hypertension and dyslipidemia, collectively called metabolic syndrome (MetS), whose pathogenesis involves both genetic and acquired factors [1]. Indeed, the pathogenesis and pathophysiology of T2DM are driven by complex interactions between the genetic predisposition and lifestyle acting on the status of metabolic health [2]. According to the World Health Organization (WHO), MetS was defined as the presence of IR (impaired fasting glucose, impaired glucose tolerance or T2DM) in addition to two of the following risk factors: obesity (waist–hip ratio or body mass index [BMI]), hyperlipidemia (hypertriglyceridemia, low high-density lipoprotein [HDL] cholesterol), hypertension or microalbuminuria [3].

Insulin resistance (IR) is one of the typical risk factors that can lead to T2DM. IR is characterized by derangements in insulin-mediated blood glucose management and the use of blood glucose, abnormal lipid deposition and increased lipid decomposition activities in adipocytes [4]. IR is one of the typical characteristics of MetS, preceding the occurrence of T2DM, metabolic dysfunction-associated steatotic liver disease (MASLD), tumors, cardiovascular disease and other metabolic diseases. It is also considered a risk factor for developing cardiovascular diseases. It arises from diverse factors, such as genetics, obesity, chronic inflammation or infection [4,5,6]. The early emergence of IR in obese individuals and its association with the development of diabetes have been well described in the literature [7,8].

Despite epidemiological studies showing an association between inflammation and T2DM, or obesity, little is known about the exact underlying pathophysiological mechanisms [9,10]. Elevated inflammatory markers and mediators and acute-phase reactants, such as fibrinogen, C-reactive protein (CRP), interleukin (IL)-6, plasminogen activator inhibitor-1 (PAI-1), sialic acid and white cell counts, have been reported to have a relationship with incident T2DM [11,12,13,14]. Thus, the above-published studies have proposed a possible association between IR and inflammation in the pathogenesis of T2DM [15,16].

It has been reported that IR is mainly involved in the pathogenesis and pathophysiology of metabolism-related diseases, such as diabetes mellitus, hypertension, tumors and MASLD [4]. Moreover, previous studies have shown a significant correlation between high-sensitivity CRP (hsCRP) and components of MetS [17,18]. Along the continuum of these previous literatures, we conducted this single-center, retrospective, cohort study to examine whether IR and hsCRP have a relationship with metabolic abnormalities in Korean patients with T2DM.

## 2. Patients and Methods

### 2.1. Study Patients and Setting

The current study was conducted on a total of 7109 Korean patients with T2DM who visited our hospital in Korea between January 2012 and December 2021.

Inclusion criteria for the current study are as follows:

(1) Korean patients with a confirmed diagnosis of T2DM [19]

(2) Patients with IR [20].

Exclusion criteria for the current study are as follows:

(1) Patients receiving insulin therapy

(2) Patients with lost-to-follow-up.

We therefore enrolled a total of 3758 patients (*n* = 3758) with T2DM in the current study; it was approved by the Institutional Review Board (IRB) of the Internal Institutional Review Board (IRB) of the Korea National Institute of Bioethics Policy (IRB approval #: P01-202201-02-003). Informed consent was waived because of the retrospective nature of the current study according to the ethical guidelines of the Korea National Institute of Bioethics Policy.

### 2.2. Data Collection

The patients were placed in a resting position for more than 5 min and then underwent blood pressure measurement using an automated sphygmomanometer (SureSigns VS3; Philips, North Ryde, Australia). Thus, their systolic and diastolic blood pressure (SBP and DBP) were measured.

Body mass index (BMI) was calculated by dividing the body weight by standing height squared (kg/m^2^). Carotid atherosclerosis was defined as the presence of isolated focal plaque or mean intima–media thickness (IMT) of >1.0 mm [21].

Blood samples were obtained from each patient in the morning after overnight fasting. Moreover, serum CRP levels were measured using a highly-sensitive sandwich enzyme-linked immunosorbent assay (ELISA) technique using anti-human-CRP goat antibody (primary one) and rabbit one (secondary one), horseradish peroxidase-conjugated anti-rabbit-IgG goat IgG (tertiary one). Furthermore, the sensitivity of the assay and the inter- and intra-assay variations were set at 0.2 µg/L and 2.5 and 5.0%, respectively [22].

### 2.3. Assessment of IR

The euglycemic hyperinsulinemic clamp has been considered the “gold standard” technique for the assessment of in vivo insulin action. It remains problematic, however, as its demerits include sophisticated equipment, relatively longer procedure time and considerable expense [23]. In contrast, the insulin tolerance test (ITT) is a simpler, easier and more practical method compared to the euglycemic hyperinsulinemic clamp [24]. The K index of the insulin tolerance test (KITT) (%/min) is used to calculate the glucose disappearance rate (GDR), which has a close association with glucose clamp studies [25].

In the current study, IR was assessed using the KITT. The GDR was calculated as previously described [26]. The KITT ranged between 0.693 and t_1/2_, where t_1/2_ is the length of time required to lower baseline glucose levels by half. The t_1/2_ value was calculated using the slope of least square analysis of the glycemic concentrations, starting at the 3rd minute until the 15th minute after a regular intravenous insulin injection (0.1 U/kg). for the current study, the state of IR was defined as the values of the KITT < 2.5%/min, as previously described [27].

The patients visited us after more than 10 h of fasting. Blood samples were collected through the placement of a 20-G catheter in the antecubital vein of the unilateral arm. Insulin, or glucose after the ITT, was injected through a placement of a 20-G catheter in the antecubital vein of the contralateral arm. The patients were given 0.1 U/kg insulin (Humulin R; Eli Lilly, Indianapolis, IN, USA) at resting state, accompanied by the collection of blood samples at a 3-min interval for a maximum period of 15 min. Immediately after the ITT, the patients were given 20% glucose 100 mL. This was followed by the centrifugation of blood samples and the measurement of plasma glucose levels using the glucose oxidase method with the Beckman glucose analyzer II (Beckman Instruments, Fullerton, CA, USA) [28].

### 2.4. Patient Evaluation and Criteria

Baseline characteristics of the patients include age, sex, duration of T2DM, systolic blood pressure (SBP), diastolic blood pressure (DBP), waist circumference, body mass index (BMI), visceral fat thickness (VFT), fasting plasma insulin levels, C-peptide levels, glycated hemoglobin (HbA1c), fasting plasma glucose (FPG), postprandial plasma glucose (PPG), homeostatic model assessment of IR (HOMA-IR), homeostatic model assessment of β-cell function (HOMA-β), aspartate aminotransferase (AST), alanine aminotransferase (ALT), total cholesterol (TC), triglyceride (TG), high-density lipoprotein cholesterol (HDL-C), low-density lipoprotein cholesterol (LDL-C), albuminuria, intima-media thickness (IMT) and hsCRP.

The patients were stratified according to the tertile of KITT or hsCRP. Thus, they were divided into the lowest (≥2.37), middle (1.54–2.36) and highest tertile (0–1.53) of KITT and the lowest (0.00–0.49), middle (0.50–1.21) and highest tertile (≥1.22) of hsCRP. Differences in baseline characteristics of the patients depending on the tertile were analyzed. Moreover, associations of KITT and hsCRP with metabolic abnormalities, such as steatotic liver disease (SLD), MetS, albuminuria and diabetic retinopathy, were also analyzed.

### 2.5. Statistical Analysis

All data was expressed as mean ± SD (SD: standard deviation). Statistical analysis was performed using the SPSS 17.0 for Windows (SPSS Inc., Chicago, IL, USA). Prior to the analysis of the patient data, we analyzed the normality of the data distribution using the Shapiro–Wilk test. To examine whether both IR and hs-CRP had a significant effect on metabolic abnormalities, we performed a logistic regression analysis and calculated odds ratios (ORs) with 95% confidence intervals (CIs). A *p*-value of <0.05 was considered statistically significant.

## 3. Results

### 3.1. Baseline Characteristics of the Patients

The study population comprises a total of 3758 Korean patients with T2DM (1382 men and 2376 women; mean age = 57.60 ± 10.26 [range, 47–67] years old). Baseline characteristics of the patients are represented in Table 1.

### 3.2. Associations of IR and hsCRP with Metabolic Abnormalities

**Figure 1:**
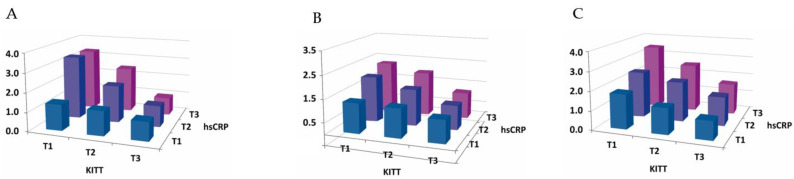
Prevalence of metabolic abnormalities depending on the tertile of KITT. (**A**) Steatotic liver disease and (**B**) albuminuria showed a significant increase from the lowest to highest tertile of KITT and hsCRP. (**C**) Metabolic syndrome showed a significant increase from the lowest to highest tertile of hsCRP (all *p* for trend < 0.001).

SLD, MetS, albuminuria and diabetic retinopathy showed a significant increase from the lowest to highest tertile of KITT (*p* for trend < 0.001). Moreover, SLD, MetS and albuminuria showed a significant increase from the lowest to highest tertile of hsCRP (*p* for trend < 0.001) (Figure 1) (Table 2).

The ORs with 95% CI for the association between insulin sensitivity and metabolic abnormalities from the lowest to highest tertile of KITT (SLD, metabolic syndrome, albuminuria and diabetic retinopathy) include 2.499 (1.968–3.714), 2.971 (2.520–3.503), 3.098 (2.605–3.684) and 2.066 (1.514–2.819), respectively (*p* for trend < 0.001). Moreover, the ORs with 95% CI for the association between hsCRP and metabolic abnormalities from the lowest to highest tertile of hsCRP (SLD, metabolic syndrome and albuminuria and diabetic retinopathy) include 2.602 (2.190–3.091), 2.782 (2.360–3.278) and 1.738 (1.470–2.056), respectively (*p* for trend < 0.001) (Table 3).

## 4. Discussion

In the current study, we found significant positive associations between the prevalence of SLD, MetS, albuminuria and diabetic retinopathy with KITT (*p* < 0.001). Our results also showed significant positive associations between the prevalence of SLD, MetS and albuminuria with hsCRP (*p* < 0.001). These results are in agreement with previously published literature showing positive relationships between hsCRP levels with IR and MetS [29,30,31,32].

An essential element of T2DM, IR is associated with metabolic abnormalities [33,34]. This is also associated with an increased risk of cardiovascular disease [28,35]. It can therefore be inferred that accurate assessment of IR in patients with T2DM is essential for not only providing the most suitable treatment modalities for them but also controlling the risk of cardiovascular disease [36,37,38].

Severe metabolic impairment occurs as a result of altered glucose and lipid metabolism; there are numerous complex interactions between the liver and other endocrine organs [39]. Evidence suggests that endocrine dysregulation has both direct and indirect effects on the pathogenesis of hepatic diseases and their severity [40]. It is well known that patients with T2DM are more vulnerable to MASLD and they have a higher risk of developing hepatic fibrosis or liver cirrhosis as compared to non-diabetic individuals [41]. There is a strong association between MASLD and IR [42]. In more detail, there is an interplay between MASLD, obesity and IR; the pathogenesis of MASLD primarily involves IR. Obesity is closely associated with IR, and it significantly raises the risk of MASLD, with approximately 30–90% of patients with obesity developing hepatic steatosis [43]. Moreover, patients with MASLD have impaired insulin suppression of free fatty acids (FFA) as well as an inhibition of fatty acid oxidation. Therefore, MASLD is closely associated with a decrease in the uptake and use of glucose as a body fuel [44]. Taken together, these findings suggest that IR may serve as an intrinsic factor involved in MASLD [45].

Despite the presence of experimental studies indicating that there is an interaction between IR and albuminuria, there is clinical evidence supporting that IR might serve as an indicator of microalbuminuria [46,47,48,49,50,51,52]. Presumably, this might be because insulin signaling is an essential element for the functions of podocytes in the glomerular filtration barrier [49,50,51,52]. Moreover, experimental studies have also suggested that IR and hyperglycemia might be associated with malfunction and loss of podocytes and thereby involved in the pathophysiological process of diabetic nephropathy and albuminuria [49,50,51,52].

Evidence shows that IR is an independent predictor of cardiovascular disease and mortality in diabetic patients and diabetic retinopathy is associated with increased cardiovascular morbidity and mortality, albuminuria and other features of metabolic syndrome. It can therefore be inferred that diabetic retinopathy may occur in association with IR [53,54,55].

It has been reported that patients with SLD have elevated levels of inflammatory markers [56]. Of inflammatory markers, hsCRP is an acute-phase reactant that serves as a non-specific marker of low-grade inflammation. Its association with metabolic syndrome or arteriosclerosis has been well described in the literature [57,58]. Its serum levels are elevated in individuals with obesity, dyslipidemia or hyperglycemia, all of which constitute the characteristics of metabolic syndrome [59]. Still, however, controversial opinions exist regarding the relationship between hsCRP and SLD [60,61,62,63].

Tsioufis C, et al. and Pedrinelli R, et al. showed that hsCRP levels were elevated in non-diabetic patients with untreated essential hypertension accompanied by microalbuminuria as compared to those with normal albumin-to-creatinine ratio [64,65]. Presumably, this might be because subclinical inflammation arising from damage to the kidney may affect glomerular functions, thus being involved in the occurrence of microalbuminuria [66,67,68,69].

According to a review of the literature, hsCRP had a significant correlation with other disease conditions, such as diabetic nephropathy, cancer and atherosclerosis [70,71,72]. Moreover, T2DM also had a significant correlation with inflammation [73]. This deserves further study.

The possible association of IR and hsCRP with metabolic abnormalities, collectively called MetS, in the context of T2DM deserves special attention. It is presumed that MetS has a relationship with chronic low-grade inflammatory reactions that are characterized by overt cytokine production and inflammatory signaling pathways [74]. Moreover, it is also believed that MetS has a relationship with obesity, adipokines and IR [75,76,77,78,79]. A key marker of systemic inflammation and a predictor of T2DM, hsCRP has been of increasing interest; the association of hsCRP with MetS and its individual components has been well described in the literature [80]. Still, however, controversial opinions exist regarding the causal relationships between them.

The possible association of IR and hsCRP with vascular endothelial dysfunction also deserves special attention. IR is mainly involved in the pathophysiology of metabolic alterations and endothelial dysfunction. Adults with hyperglycemia are characterized by peripheral IR consisting of impaired insulin action on peripheral tissues [4]. IR is a key player in the context of MetS; it has a possible association with an increased release of circulating FFA from the expanded adipose tissue [81]. FFA inhibits insulin-mediated glucose uptake in muscles, elevating circulating glucose and promoting pancreatic insulin secretion. In the liver, FFA promotes the production of glucose, TG and very LDL and inhibits the transformation of glucose into glycogen [82]. IR promotes lipolysis of stored triacylglycerol in the adipose tissue and the synthesis of FFA because insulin inhibits lypolysis [83]. As a result, hypertriglyceridemia contributes to the reduction in the cholesteryl ester content in the lipoprotein core and consequently in protective HDL [84]. Moreover, the relative depletion of unesterified and esterified cholesterol and phospholipids contributes to the formation of LDL [84]. IR is also associated with elevated levels of apo B and C-III, uric acid and prothrombotic factors, which results in endothelial dysfunction and vascular remodeling [84]. Finally, hsCRP is considered both a biomarker of the process of endothelial dysfunction and a predictor of vascular disease [85].

To summarize, our results are as follows:

1. There was a significant positive association between the prevalence of SLD, MetS, albuminuria and diabetic retinopathy and KITT (*p* < 0.001).
2. There was a significant positive association between the prevalence of SLD, MetS and albuminuria and hsCRP (*p* < 0.001).

Our findings cannot be generalized because there are three limitations of the current study: First, we performed a retrospective review of the medical records of the patients who had been treated at a single local clinic. The possibility of selection bias could not therefore be completely ruled out. Second, we failed to perform causal relationships of IR and hsCRP with metabolic abnormalities in the context of T2DM. Third, we failed to consider sex differences in assessing associations of IR and hsCRP with metabolic abnormalities in Korean patients with T2DM. Sex difference also plays a role in developing IR; IR is more commonly seen in men compared to women because of elevated visceral and hepatic adipose tissue with a lack of a possible protective effect of estrogen and lower adiponectin levels. This explains why men are at a higher risk of T2DM compared to women [86].

## 5. Conclusions

In conclusion, our results indicate that clinicians should consider the relationships of IR and hsCRP with metabolic abnormalities in the management of patients with T2DM. However, further large-scale, prospective, multi-center studies are warranted to confirm our results.

## Figures and Tables

**Table 1:** Baseline characteristics of subjects (*n* = 3758) depending on the tertile of KITT.

Variables	Values			*p* for Trend
	Tertile 1 (*n* = 1255)	Tertile 2 (*n* = 1267)	Tertile 3 (*n* = 1236)	
KITT	3.18 ± 0.67	1.93 ± 0.23 ^a^	1.08 ± 0.31 ^a,b^	<0.001
Age (years old)	56.47 ± 9.95	57.86 ± 10.12 ^a^	58.46 ± 10.71 ^a^	<0.001
Duration (years)	6.94 ± 6.85	7.63 ± 7.23 ^a^	8.68 ± 7.30 ^a,b^	<0.001
Sex (men, %)	669 (53.3%)	637 (50.3%)	646 (52.3%)	
SBP (mmHg)	132.08 ± 16.63	136.97 ± 17.95 ^a^	138.02 ± 18.94 ^a^	<0.001
DBP (mmHg)	84.18 ± 11.06	86.24 ± 11.48 ^a^	87.09 ± 11.82 ^a^	<0.001
Waist (cm)	81.42 ± 8.15	83.99 ± 8.18 ^a^	86.31 ± 8.56 ^a,b^	<0.001
BMI (kg/m2)	23.90 ± 2.89	24.69 ± 2.96 ^a^	25.36 ± 3.43 ^a,b^	<0.001
VFT (mm)	39.95 ± 16.47	45.61 ± 17.30 ^a^	50.20 ± 18.78 ^a,b^	<0.001
Fasting plasma insulin (nmol/IU)	7.38 ± 3.91	8.53 ± 4.30 ^a^	10.20 ± 5.47 ^a,b^	<0.001
C-peptide (nmol/L)	1.69 ± 0.72	1.96 ± 0.79 ^a^	2.31 ± 1.02 ^a,b^	<0.001
HbA1c (%)	7.65 ± 1.61	8.20 ± 1.82 ^a^	8.99 ± 2.06 ^a,b^	<0.001
FPG (mg/dL)	138.31 ± 41.06	154.25 ± 52.30 ^a^	185.20 ± 67.18 ^a,b^	<0.001
PPG (mg/dL)	201.62 ± 80.96	227.40 ± 84.30 ^a^	271.57 ± 91.29 ^a,b^	<0.001
HOMA-IR	2.49 ± 1.45	3.17 ± 1.80 ^a^	4.53 ± 2.84 ^a,b^	<0.001
HOMA-β	49.85 ± 66.66	48.76 ± 52.30	43.08 ± 47.96 ^a,b^	<0.006
AST (U/L)	25.00 ± 10.21	26.39 ± 12.30 ^a^	30.30 ± 16.38 ^a,b^	<0.001
ALT (U/L)	25.81 ± 16.93	29.10 ± 19.38 ^a^	34.49 ± 25.39 ^a,b^	<0.001
Total cholesterol (mg/dL)	189.82 ± 38.41	198.33 ± 38.24 ^a^	201.59 ± 43.96 ^a^	<0.001
TG (mg/dL)	121.74 ± 73.54	150.22 ± 97.37 ^a^	183.62 ± 136.10 ^a,b^	<0.001
HDL-C (mg/dL)	51.38 ± 13.47	50.09 ± 13.12 ^a^	48.24 ± 12.75 ^a,b^	<0.001
LDL-C (mg/dL)	112.13 ± 32.64	116.95 ± 33.02	117.47 ± 36.41 ^a^	<0.001
Albuminuria (g/L)	37.57 ± 51.90	48.42 ± 56.68 ^a^	69.30 ± 68.60 ^a,b^	<0.001
IMT	0.82 ± 0.17	0.84 ± 0.18 ^a^	0.85 ± 0.18 ^a^	<0.001
Max IMT (mm)	0.89 ± 0.20	0.91 ± 0.21 ^a^	0.92 ± 0.21 ^a^	<0.001
CRP (mg/L)	1.47 ± 3.48	1.78 ± 4.72	2.72 ± 7.61 ^a,b^	<0.001

Abbreviations: KITT, K index of the insulin tolerance test; SBP, systolic blood pressure; DBP, diastolic blood pressure; BMI, body mass index; VFT, visceral fat thickness; FPG, fasting plasma glucose; PPG, postprandial plasma glucose; HOMA, homeostatic model assessment; IR, insulin resistance; AST, aspartate aminotransferase; ALT, alanine aminotransferase; TG, triglyceride; HDL-C, high-density lipoprotein cholesterol; LDL-C, low-density lipoprotein cholesterol; IMT, intima–media thickness. Values are mean ± standard deviation or number with percentage, where appropriate. ^a^ *p* < 0.05 vs. T1, ^b^ *p* < 0.05 vs. T2.

**Table 2:** Prevalence of metabolic abnormalities depending on tertile of KITT and hsCRP.

Variables		Values			*p* for Trend
		Tertile 1 (*n* = 1255)	Tertile 2 (*n* = 1267)	Tertile 3 (*n* = 1236)	
**KITT**					
	Steatotic liver disease	448 (24.40%)	645 (35.10%)	743 (66.20%)	<0.001
	Metabolic syndrome	424 (11.50%)	606 (16.50%)	739 (20.10%)	<0.001
	Albuminuria	342 (30.40%)	505 (43.80%)	653 (57.50%)	<0.001
	Diabetic retinopathy	65 (5.30%)	102 (8.20%)	126 (10.50%)	<0.001
**hsCRP**					
	Steatotic liver disease	446 (41.80%)	647 (58.50%)	743 (65.20%)	<0.001
	Metabolic syndrome	411 (34.10%)	626 (50.80%)	732 (59.00%)	<0.001
	Albuminuria	401 (36.50%)	510 (44.90%)	589 (50.00%)	<0.001
	Diabetic retinopathy	115 (9.40%)	93 (7.70%)	85 (6.90%)	<0.020

Abbreviations: KITT, K index of the insulin tolerance test; hsCRP, high-sensitivity C-reactive protein. Values are number with percentage.

**Table 3:** Associations of KITT and hsCRP with metabolic abnormalities.

Variables		Values				
		Tertile 1 (*n* = 1255)	Tertile 2 (*n* = 1267)		Tertile 3 (*n* = 1236)	
		OR (95% CI)	OR (95% CI)	*p*	OR (95% CI)	*p*
**KITT**						
	Steatotic liver disease	1.00	1.756 (1.409–2.189)	<0.001	2.499 (1.968–3.714)	<0.001
	Metabolic syndrome	1.00	1.836 (1.561–2.159)	<0.001	2.971 (2.520–3.503)	<0.001
	Albuminuria	1.00	1.782 (1.500–2.117)	<0.001	3.098 (2.605–3.684)	<0.001
	Diabetic retinopathy	1.00	1.580 (1.146–2.180)	<0.005	2.066 (1.514–2.819)	<0.001
**hsCRP**						
	Steatotic liver disease	1.00	1.960 (1.652–2.324)	<0.001	2.602 (2.190–3.091)	<0.001
	Metabolic syndrome	1.00	1.995 (1.694–2.349)	<0.001	2.782 (2.360–3.278)	<0.001
	Albuminuria	1.00	1.416 (1.195–1.678)	<0.001	1.738 (1.470–2.056)	<0.001
	Diabetic retinopathy	1.00	0.795 (0.597–1.058)	<0.116	0.710 (0.530–0.950)	<0.021

Abbreviations: KITT, K index of the insulin tolerance test; hsCRP, high-sensitivity C-reactive protein.

## Data Availability

The data presented in this study are available on request from the corresponding author. The data are not publicly available due to privacy reasons.
